# Impact of Chemotherapy on Vaccine Immunogenicity and Revaccination Response of Acute Lymphoblastic Leukemia—A Systematic Review and Meta-Analysis

**DOI:** 10.3390/vaccines13060605

**Published:** 2025-06-01

**Authors:** Yuyuan Zeng, Chuanyu Yang, Xihan Li, Qi An, Bo Zhou, Wenquan Niu, Yu Tian, Yifei Cheng, Lin Wang

**Affiliations:** 1 Capital Institute of Pediatrics, Chinese Academy of Medical Sciences & Peking Union Medical College, Beijing 100020, China; 13365065979@163.com (Y.Z.); yangchuanyu1234@163.com (C.Y.); 2 Department of Child Health Care, Capital Center for Children’s Health, Capital Medical University, Beijing 100020, China; anqi20190220@163.com (Q.A.); bobo-0207@hotmail.com (B.Z.); yut262@126.com (Y.T.); 3 School of Public Health, Capital Medical University, Beijing 100069, China; ethanpage@163.com; 4 Center for Evidence-Based Medicine, Capital Center for Children’s Health, Capital Medical University, Beijing 100020, China; niuwenquan_shcn@163.com; 5 Peking University Institute of Hematology, Peking University People’s Hospital, Peking University, Beijing 100044, China

**Keywords:** chemotherapy, vaccine, antibody, immunogenicity, revaccination response, acute lymphoblastic leukemia

## Abstract

Background: Chemotherapy, a cornerstone treatment for Acute Lymphoblastic Leukemia (ALL), can compromise immune function, leading to impaired immune memory function and diminished responses to revaccination. This systematic review and meta-analysis sought to evaluate the impact of chemotherapy on the immunogenicity of prior vaccinations and subsequent revaccination responses in children with ALL. Methods: A comprehensive search was conducted through PubMed, Embase, Web of Science, and Medline. Search time was 9 January 2025. R 4.4.2 was employed for data analysis. Results: A total of 29 relevant studies were identified, with 8 undergoing meta-analysis. The pooled antibody seropositive rates (SPR) for vaccines against Hepatitis B Virus (HBV), Hepatitis A Virus (HAV), diphtheria, tetanus, pertussis, measles, mumps, rubella, varicella, and Pneumococcal Conjugate Vaccine (PCV) demonstrated a statistically significant decline after chemotherapy in ALL patients (*p* < 0.0001). Subgroup analysis further revealed marked and heterogeneous declines in SPR after chemotherapy, with the magnitude of reduction varying significantly across vaccines—tetanus, HBV, HAV, measles, mumps, and rubella (Subgroup differences, *p* = 0.0037). Conclusions: This review provides an updated assessment of this critical topic, representing the first meta-analysis specifically focused on the effects of chemotherapy on different vaccines’ immunogenicity in children with ALL.

## 1. Introduction

Acute lymphoblastic leukemia (ALL) is a malignant disease arising from a complex interplay of genetic predisposition and environmental factors, representing the most prevalent malignancy in childhood. Accounting for approximately two-thirds of all acute leukemias, ALL is characterized by genetic mutations affecting lymphoid development, differentiation, and cell-cycle regulation, often accompanied by chromosomal abnormalities, leading to uncontrolled lymphocyte proliferation [1,2]. Clinical manifestations of ALL typically include fever, fatigue, bruising, bleeding, and lymphadenopathy. Diagnosis of ALL is based on the 2022 World Health Organization (WHO) classification guidelines, which integrate the characterization of cell morphology, immunophenotypes, molecular biology, and cytogenetics [3].

The first-line regimen is chemotherapy for over 2–3 years, encompassing induction, consolidation, intensive, and maintenance therapy [3,4]. These regimens commonly include glucocorticoids, vincristine (VCR), asparaginase, anthracyclines, cyclophosphamide, methotrexate (MTX), cytarabine, and mercaptopurine, among others. After treatment, the current 5-year disease-free survival rate of children with ALL can reach 90% [5,6]. Chemotherapy drugs primarily exert their effects by disrupting DNA structure or inhibiting DNA replication [7], interfering with enzyme and protein synthesis, damaging cellular structural components, and inhibiting tumor angiogenesis. Consequently, these cytotoxic processes inevitably impact protective immune cells. Vaccination is a cornerstone of preventative medicine, inducing an immune response to specific antigens and generating memory B and T cells, thereby providing long-lasting protection. The detrimental effects of chemotherapy on vaccine-induced immune memory function may compromise this preventative efficacy.

Numerous articles have been published regarding the influence of chemotherapy on the immunogenicity of vaccines in patients with ALL. Previously, a review of summary vaccination data, including antibody titers of diphtheria, B. pertussis, tetanus, poliomyelitis, and Haemophilus influenzae type b (Hib) in children with ALL, incorporating articles published between 1980 and 2006. Recent research has expanded upon these findings, incorporating a broader range of vaccines and immunological indicators. Therefore, this review aims to systematically evaluate the updated literature through meta-analysis to comprehensively assess the impact of chemotherapy on vaccine immunogenicity and revaccination responses in ALL patients.

## 2. Materials and Methods

### 2.1. Search Strategy

Following the Preferred Reporting Items for Systematic Reviews and Meta-Analyses (PRISMA) guidelines [8], the systematic review systematically evaluated the impact of chemotherapy on vaccine immunogenicity and revaccination responses in ALL patients. A comprehensive literature search was conducted in the databases of PubMed, Embase, Web of Science, and Medline with no restriction on the year of publication. The keywords used for database searches for this review were (‘Vaccine’ OR ‘Vaccines’ OR ‘Vaccinate’ OR ‘Vaccination’) AND (‘immunization’ OR ‘immunogenicity’ OR ‘immunity’ OR ‘Antibodies’ OR ‘Antibody’ OR ‘Antigenicity’ OR ‘immune response’) AND (‘Acute Lymphocytic Leukemia’ OR ‘Acute Lymphocytic Leukemia’ OR ‘Acute Lymphoblastic Leukemia’ OR ‘Acute Lymphoid Leukemia’). Search time was 9 January 2025. Additionally, the systematic review methods were established and registered a priori in PROSPERO (CRD42024621362).

### 2.2. Eligibility Criteria

The PICO framework was used to develop eligibility criteria for this review. The ‘population’ comprised individuals diagnosed with ALL who received any vaccine according to local vaccination policy and were younger than 18 years old at the time of diagnosis. The ‘intervention’ was ‘chemotherapy’, administered according to standard regimens. The ‘Comparator’ included ALL patients prior to chemotherapy initiation and healthy individuals vaccinated with the same vaccines. The ‘outcome’ was the immunogenicity of vaccines assessed by geometric mean concentration (GMC), geometric mean titer (GMT), and seropositivity rate (SPR). Eligible study designs included original research articles; case reports, reviews, meta-analyses, and letters were excluded.

### 2.3. Exclusion Criteria

Studies were excluded if they involved ALL patients who received immunotherapy, radiotherapy, hematopoietic stem cell transplantation, or bone marrow transplantation in addition to chemotherapy. Studies with uncertain vaccination records were also excluded. Articles containing useful data meeting the criteria and detailed data of patients would be included and the useful information would be extracted, even if the entire article was not fully eligible. Articles published in languages other than English were excluded.

### 2.4. Data Extraction

A data extraction form was developed for this systematic review. The extraction process was piloted independently by two reviewers to ensure the quality and consistency of data collection. Reviewers independently extracted data from each article, and conflicts were resolved by discussion. The following data were extracted from each study: first author, study setting, year of publication, study design, sample size, chemotherapy regimen, population characteristics, type of comparator, vaccine(s) studied, revaccination status, sampling time points, and key findings related to immunogenicity. The primary effect measures extracted were GMT, GMC, SPR, and corresponding odds ratio (OR) and 95% confidence interval (CI).

### 2.5. Quality Assessment

Following data extraction, two reviewers independently assessed the risk of bias within the study using the JBI Critical Appraisal Checklist for Analytical Cross-Sectional Studies. Disagreements were resolved through discussion and consensus.

### 2.6. Statistical Analysis

R 4.4.2 (Vienna, Austria) was employed for data analysis. Heterogeneity between studies was assessed using the Q-test. For those with a high degree of heterogeneity, the random-effects model will be adopted, while for those with a low degree of heterogeneity, the fixed-effects model will be used. Publication bias was evaluated using Egger’s and Begg’s tests. Subgroup analysis was used to explore possible causes of heterogeneity.

## 3. Result

### 3.1. Literature Search Results

**Figure 1:**
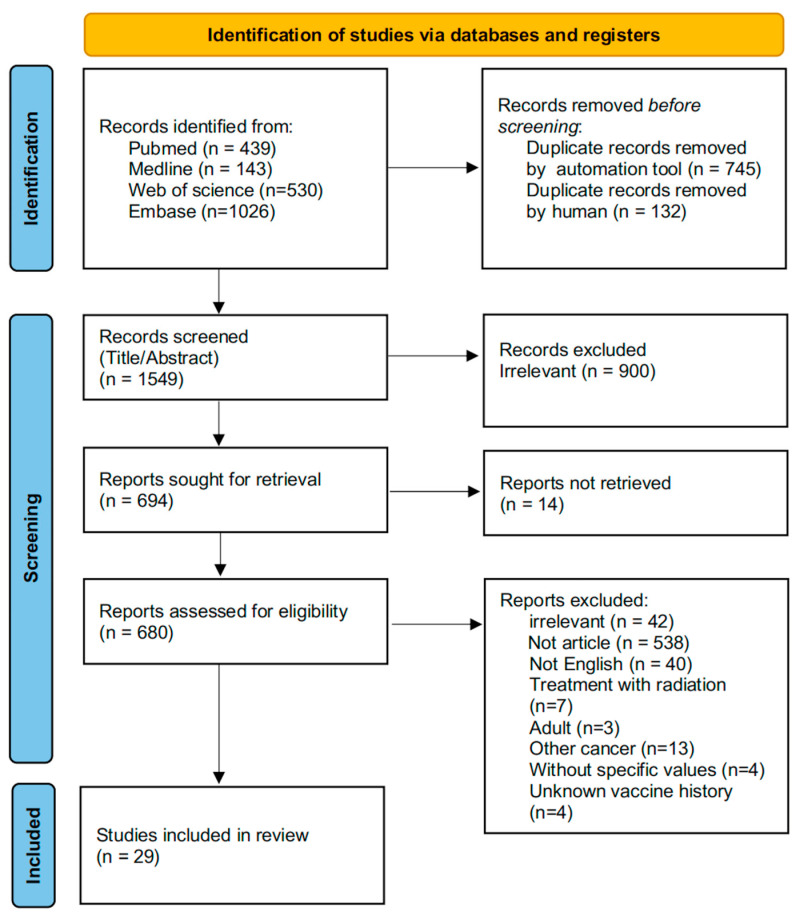
A PRISMA flow diagram of information from identification to inclusion.

A total of 2138 studies were retrieved after an initial search of PubMed, Web of Science, Embase and Medline. Duplicate records were removed using an automation tool (*n* = 745) and manual screening (*n* = 132). Following reviews of titles and abstracts, 900 records with irrelevant topics were excluded. Based on the full text review, 29 articles were finally selected for further analysis (Figure 1).

### 3.2. Characteristics of Identified Studies

The included studies with a total of 2556 participants were published between 1971 and 2025, the studies evaluated the immunogenicity of 15 different vaccines: measles (*n* = 5), mumps (*n* = 4), rubella (*n* = 6), poliovirus (*n* = 3), haemophilus influenzae B (*n* = 5), varicella (*n* = 3), diphtheria (*n* = 4), tetanus (*n* = 7), pertussis (*n* = 2), hepatitis B virus (*n* = 7), hepatitis A virus (*n* = 2), pneumococcus (*n* = 3), and influenza H1N1 (*n* = 6), influenza H3N2 (*n* = 3), influenza B (*n* = 2) (Table 1). Twenty-three studies assessed the efficacy of booster immunization, while seven articles focused solely on comparing immunogenicity pre-chemotherapy, post-chemotherapy, and healthy controls. The study populations included patients in the newly diagnosed period, the maintenance treatment period, or on cessation of chemotherapy. They had received age-appropriate vaccinations according to their respective national immunization programs, including those established by the U.S. Centers for Disease Control (CDC), the Finnish National Vaccination Program, the Swedish Public Health Agency, and so on. In addition to GMC, GMT, and SPR, lymphocyte subsets, immunoglobulin subclasses, and lymphocyte proliferation assay were also evaluated in several studies. One study published in 2020 discovered that 27–30% of patients within 4–12 months of completing chemotherapy had total CD3+ T cells, CD4+ T cells, and CD8+ T cells below the 10th percentile for age [9]. Lehrnbecher et al. found no differences in CD3+ T cells, CD4+ T cells, and CD8+ T cells, and immunoglobulin subclasses among patients at 3, 6, and 9 months after completion of chemotherapy [10].

### 3.3. Immunogenicity Variation After Chemotherapy

A total of eight articles, encompassing 10 vaccine types (HBV, HAV, measles, mumps, rubella, varicella, diphtheria, tetanus, pertussis, and PCV), compared SPR values in ALL patients before chemotherapy or in healthy controls with ALL patients after chemotherapy [9,11,12,13,14,15,16,17]. These studies consistently demonstrated a decline in SPR values for all vaccines examined. Five studies evaluated GMT for five different vaccines: poliovirus, influenza B, H3N2, H1N1, and HBV [14,18,19,20,21]. Two studies involving poliovirus and HBV showed a decrease in GMT values after chemotherapy [14,18]. The remaining three articles, focusing on influenza B, H3N2, and H1N1 subtypes, yielded conflicting results [19,20,21]. Lange et al. found no significant difference in antibody levels for influenza H3N2 and H1N1 in ALL patients undergoing maintenance therapy, those off therapy for a mean of 15 months, and sibling controls [19]. Shahgholi et al. reported that ALL patients undergoing maintenance therapy had similar antibody levels for influenza B, H3N2, and H1N1 compared to healthy controls [21]. Conversely, Porter et al. showed that GMTs of influenza H3N2 and H1N1 in ALL patients in first remission and receiving maintenance chemotherapy were significantly lower than those in healthy controls [20].

### 3.4. Revaccination Response

Ten studies, encompassing 10 vaccine types (HBV, Hib, diphtheria, tetanus, pertussis, measles, mumps, rubella, PCV, and influenza H1N1), compared SPR values before and after revaccination in ALL patients who had completed or were receiving chemotherapy [10,12,22,23,24,25,26,27,28,29]. Two studies evaluating Hib revealed a significant increase in SPR values 4–6 weeks after revaccination (*p* < 0.001) and 3 months after revaccination, respectively (*p* < 0.05) [10,22]. Three studies reported increases in SPR values for diphtheria, ranging from 13/31 to 25/31, 7/21 to 21/21, and 41/75 to 56/60 [10,12,24]. Three articles focused on antibody levels of tetanus, demonstrating increases of 35.5–57.1% [10,12,24]. SPR values for measles increased from 32/77 to 53/68, 14/75 to 25/45, and 43/57 to 51/57; for rubella, from 57/77 to 69/70, 31/75 to 36/45, and 43/57 to 54/57; and for mumps, from 41/77 to 60/70, 25/75 to 38/45, and 41/57 to 55/57 across three studies [12,25,27]. SPR values for PCV increased from 2/118 to 48/113 and 27/71 to 70/71 [28,29]. One paper examined pertussis and showed an SPR increase from 0/75 to 11/60, and another study of influenza H1N1 demonstrated an SPR increase from 0/45 to 10/39 [12,26].

Lange et al. found a marked increase in GMT values for both influenza H3N2 and influenza H1N1 after revaccination in ALL patients off therapy [19]. Shahgholi et al. reached similar conclusions, studying influenza B, H3N2, and influenza H1N1, and revealing a remarkable increase in all GMT values [21]. Following revaccination, an obvious rise in GMT values for poliovirus was observed [10]. Meanwhile, they observed a notable surge in GMC values for antibodies to diphtheria, tetanus, and Hib (*p* < 0.05) [10]. A Canadian study revealed that 2 months after revaccination, GMC values to pertussis, tetanus, and PCV13 serotypes demonstrated a pronounced elevation and remained significantly above baseline levels at 12 months post-vaccination (*p* < 0.001) [9].

### 3.5. Characteristics of Studies Included in the Meta-Analysis

To account for potential publication bias when comparing GMCs and GMTs of vaccines in pre-chemotherapy versus post-chemotherapy groups, and GMCs, GMTs, and SPRs pre-revaccination versus post-revaccination (Egger’s and Begg’s tests, *p* < 0.05), a meta-analysis was performed solely on SPR data comparing values before and after chemotherapy (Egger test, *p* = 0.5012). A total of 8 studies involving HBV, HAV, diphtheria, tetanus, pertussis, measles, mumps, rubella, varicella, and PCV vaccines were included [9,11,12,13,14,15,16,17]. Several articles documented a decrease in SPR values for HBV antibody following chemotherapy. Reported decreases ranged from 34/34 to 15/34, 56/88 to 12/88, 35/46 to 11/46, 416/547 to 177/547, 88/88 to 74/88, and 65/78 to 32/78, respectively [11,13,14,15,16,17]. Toret et al. observed decreases in SPR values for measles, mumps, and rubella from 27/46, 21/46, and 11/46 at diagnosis to 11/46, 16/46, and 1/46 six months after the end of chemotherapy (*p* < 0.0001, *p* = 0.059, and *p* = 0.002, respectively) [13]. Koochakzadeh et al. compared SPR values for measles, mumps, and rubella in ALL at diagnosis with those at 1, 2, 3, 6, and 12 months after chemotherapy, finding a decrease from 7/15, 9/15, and 10/15 to 9/75, 25/75, and 28/75 [12]. Ince T. et al. also studied rubella and demonstrated a decrease from 89.7% to 48.7% (*p* < 0.001) [16]. This study further revealed a decrease in SPR values for HAV from 79.5% to 55.1% (*p* < 0.001) [15]. Another study showed a decrease in SPR values for HAV from 38/46 to 18/46 (*p* < 0.0001) [13]. Two articles reported decreases in SPR values for tetanus antibody from 14/15 and 78/78 to 36/75 and 52/74, respectively [12]. Two articles revealed that SPR values for varicella decreased from 24/46 and 41/78 to 21/56 and 15/74 [13]. Only one article involving diphtheria and pertussis found that SPR values in ALL patients at diagnosis were lower than those after chemotherapy [12].

### 3.6. Comparison of SPR Between Pre-Chemotherapy and Post-Chemotherapy

**Figure 2:**
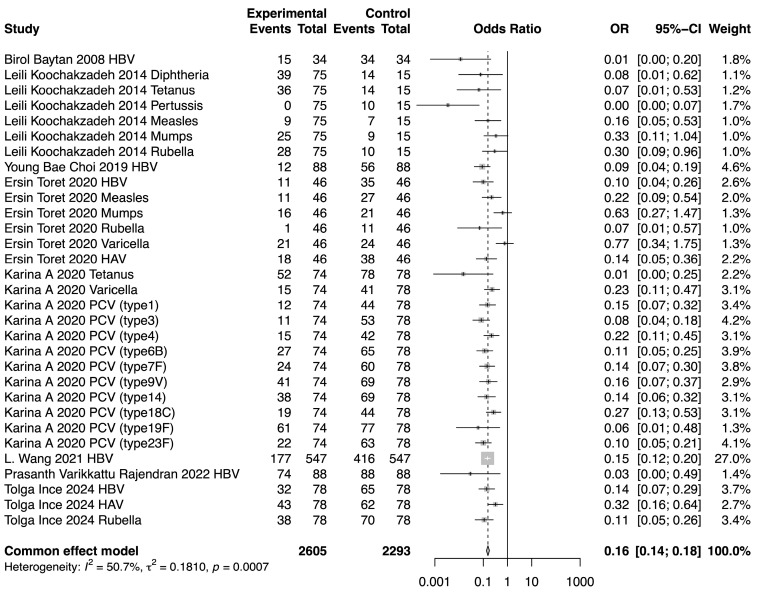
Forest plot of the pooled OR along with 95% confidence intervals [9,11,12,13,14,15,16,17].

**Figure 3:**
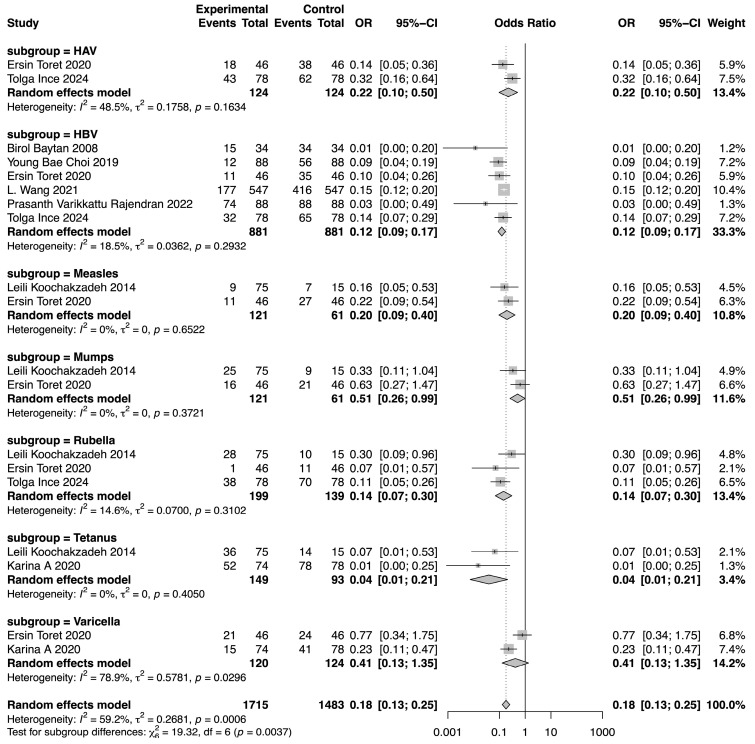
Forest plot of the pooled OR along with 95% confidence intervals in subgroups of different vaccines, including HAV, HBV, measles, mumps, rubella, tetanus, and varicella, and the subgroup differences [9,11,12,13,14,15,16,17].

The meta-analysis demonstrated a reduction in SPR values for the included vaccines in the post-chemotherapy group compared to the pre-chemotherapy group. The pooled OR for SPR values in the post-chemotherapy group versus the pre-chemotherapy group was 0.16 (95%CI 0.14–0.18, *p* < 0.0001, I^2^ = 50.7%) (Figure 2). Assessment of heterogeneity yielded an I^2^ value of 50.7%, indicating considerable heterogeneity. Hence, subgroup analysis was performed based on vaccine type, revealing that vaccine type was not the source of heterogeneity. The difference among these estimates, including tetanus, HBV, rubella, measles, HAV, mumps, and varicella, was statistically significant (χ^2^ = 19.32, *p* = 0.0037) (Figure 3). The most substantial decrease was observed for tetanus, with a pooled OR of 0.04 (95%CI 0.01–0.21, *p* = 0.0001, I^2^ = 0.0%). SPR values for HBV antibody also decreased obviously after chemotherapy, yielding a pooled OR of 0.12 (95%CI 0.09–0.17, *p* < 0.0001, I^2^ = 18.5%). The next one was rubella, for which the pooled SPR was 0.14 (95%CI 0.07–0.30, *p* < 0.0001, I2 = 14.6%). SPR of measles antibody dropped significantly due to chemotherapy with a pooled OR of 0.2 (95%CI 0.09–0.40, *p* < 0.0001, I^2^ = 0.0%), followed by HAV with a pooled OR of 0.22 (95%CI 0.10–0.50, *p* = 0.0003, I^2^ = 48.5%). Varicella and mumps were comparatively less affected by chemotherapy, the pooled ORs of which were 0.41 (95%CI 0.13–1.35, *p* = 0.14, I^2^ = 78.9%) and 0.51 (95%CI 0.26–0.99, *p* = 0.05, I^2^ = 0.0%), respectively (Figure 3).

### 3.7. Risk of Bias

The JBI Critical Appraisal Checklist for Analytical Cross-Sectional Studies was used to grade the evidence. The results of the risk of bias assessment for each included article are presented in the Supplementary Materials (Table S1).

## 4. Discussion

### 4.1. Summary of Evidence

The systematic review and meta-analysis provide an updated and comprehensive assessment of chemotherapy’s impact on vaccine immunogenicity in children with ALL. To the best of our knowledge, this is the first meta-analysis specifically focused on this population. The included studies predominantly evaluated antibody responses, with limited consideration of cellular immune function. A significant reduction in antibody SPR values was observed following chemotherapy across multiple vaccines, including those targeting HBV, measles, mumps, rubella, varicella, and HAV. The most substantial decrease was observed for the tetanus antibody, while varicella and mumps had no obvious variation, which suggested that different antibodies of vaccines exhibit varying sensitivities to chemotherapy. GMCs and GMTs of vaccines increased in all of the included studies after revaccination, indicating a positive response to booster doses. These findings suggest that children undergoing chemotherapy for ALL are at increased risk of vaccine-preventable infectious diseases and may benefit from a targeted revaccination strategy to bolster immune defenses. While a preliminary analysis revealed considerable heterogeneity, subgroup meta-analyses for individual vaccine types demonstrated lower heterogeneity in HBV, measles, mumps, rubella, and HAV, suggesting that the observed effects are relatively consistent within these specific vaccine categories. Previous studies have consistently recommended implementing systematic vaccine booster schedules for ALL patients following or during therapy, even among those who were previously fully vaccinated, to enhance protection against vaccine-preventable diseases (VPDs) [30,31,32]. These recommendations align with guidelines from the American Academy of Pediatrics, which advises revaccination for this population, specifying optimal timeframes: six months post-chemotherapy and twelve months post-stem cell transplantation [33]. The conclusions of this meta-analysis further corroborate these evidence-based revaccination strategies for ALL patients following treatment.

Previously, a systematic review published in 2006 investigated antibody loss and response to (re-)vaccination in children after treatment for ALL. This earlier review, which included only eight reports, similarly revealed a reduction in specific antibody levels, including those for diphtheria, pertussis, tetanus, poliovirus, and Hib [34]. Nevertheless, that review lacked a meta-analysis comparing patients undergoing therapy with pre-treatment or healthy controls. Our results are consistent with these earlier findings. Importantly, our study extends this previous work by directly comparing antibody SPR values in ALL patients before and after chemotherapy using meta-analysis, providing a higher level of evidence regarding the impact of chemotherapy on vaccine immunity. Furthermore, this review incorporates newly published reports since 2006 and expands the scope to include vaccines targeting measles, mumps, rubella, poliovirus, varicella, HBV, HAV, streptococcus pneumoniae, and influenza. The previous review included only a limited number of studies evaluating revaccination responses, with two studies on diphtheria, one on pertussis, two on tetanus, and two on Hib. In conclusion, our review incorporates more recent reports, providing additional information on revaccination responses and strengthening the evidence supporting the need for booster doses in this vulnerable population.

### 4.2. Limitation

This systematic review and meta-analysis acknowledge several limitations. While a total of 29 articles were included, the number of studies available for each individual vaccine was limited, reducing the statistical power for vaccine-specific analyses and compromising the validity of subgroup comparisons. Furthermore, the temporal heterogeneity in the treatment phases was substantial, encompassing both distinct stages during therapy and varying intervals following treatment completion, no matter the post-chemotherapy or post-revaccination group. Although temporal subgrouping could theoretically be attempted, vaccine types differed markedly between subgroups. Collectively, the comparability of subgroups would remain severely compromised, hindering the determination of accurate antibody levels at each stage of the process. Due to the potential for publication bias when comparing antibody levels pre- and post-revaccination, a formal meta-analysis was not performed to avoid potentially misleading conclusions. Considerable heterogeneity existed in chemotherapy administration across the study population, with variations in the number of cycles and drug dosages. This therapeutic variability may introduce confounding bias when assessing outcomes in this diverse cohort. Finally, the absence of a control group to compare revaccination responses prevented a definitive assessment of the extent to which chemotherapy compromises the immune response to vaccination in ALL patients.

### 4.3. Future Directions

The current systematic review and meta-analysis highlights a significant gap in the literature: the overwhelming focus on humoral immunity (antibody responses) in published studies regarding vaccine immunogenicity in ALL patients. Future research should prioritize investigations encompassing cellular immune functions, such as the characterization of memory T and B cell responses. This expanded approach would provide a more comprehensive profile of vaccine immunogenicity in this patient population. Additionally, prospective cohort studies involving newly diagnosed ALL patients, with close longitudinal follow-up, are warranted to better understand the impact of chemotherapy on vaccine responses and to inform optimal vaccination strategies.

## 5. Conclusions

In conclusion, our systematic review and meta-analysis provide compelling evidence of diminished antibody responses against several infectious viruses and suggest that different vaccines exhibit varying sensitivities to chemotherapy in children with ALL undergoing chemotherapy. This review underscored the importance of targeted revaccination strategies for this vulnerable patient population to mitigate the risk of vaccine-preventable infections.

## Figures and Tables

**Table 1:** Characteristics of included studies.

Author	Year	Setting	Vaccine	Subjects (N)	Control (N)	Revaccination
Pearay L. Ogra	1971	USA	Poliovirus type 1	ALL Children who had received no prior immunization with polio vaccine (10)	Healthy individuals who had received no prior immunization with polio vaccine (8)	Poliovirus type 1 (23)
				ALL who had been previously immunized with live polio vaccine (13)	Healthy individuals who had been immunized with polio vaccine (12)	
Peter A. Gross	1978	USA	Influenza H1N1	ALL on maintenance treatment period (47)	ALL not on chemotherapy for 1 month (20)	Influenza H1N1 (67)
Beverly Lange	1979	USA	Influenza H3N2	ALL on maintenance treatment period (22)	ALL not on chemotherapy for 15 months (16) and healthy siblings of ALL (22)	Influenza H3N2 (38)
			Influenza H1N1			Influenza H1N1 (38)
Mirja Stenvik	1987	Finland	Poliovirus type 1	ALL on continuous complete remission (14)	NA	Poliovirus type 1 (14)
			Poliovirus type 2			Poliovirus type 2 (14)
			Poliovirus type 3S			Poliovirus type 3S (14)
			Poliovirus type 3P			Poliovirus type 3P (14)
Beverly Lange	1989	USA	Hib	ALL on maintenance treatment period (14)	GMT = 8	Hib (14)
Allan M Arbeter	1989	USA	Oka/Merck varicella-Zoster	ALL with 6-MP while other medications were suspended for 1 week before and after vaccination (24)	ALL with chemotherapy was suspended for 1 week before and after vaccination (20)	Oka/Merck varicella-Zoster (44)
Sandor Feldman	1989	USA	Hib	Chemotherapy for over 12 months in remission, who had received no prior immunization with Hib vaccine (22)	Chemotherapy for less than 12 months on remission, who had received no prior immunization with Hib vaccine (28)	Hib (50)
Derry Ridgway	1991	Portland	Diphtheria	ALL on STD maintenance treatment period (12)	ALL on BFM maintenance treatment period (6)	Diphtheria (24)
			Tetanus		ALL on BFM maintenance treatment period (6)	Tetanus (24)
			Hib		ALL on BFM maintenance treatment period (6)	Hib (24)
			Diphtheria		ALL on NY/L maintenance treatment period (6)	Diphtheria (24)
			Tetanus		ALL on NY/L maintenance treatment period (6)	Tetanus (24)
			Hib		ALL on NY/L maintenance treatment period (6)	Hib (24)
Sevgi Yetgin	2001	Turkey	Engerix (SmithKline Beecham)	ALL on maintenance treatment period achieving complete remission with ant-HBV negative (50)	NA	Engerix (SmithKline Beecham) (50)
			GenHevac B (Pasteur Connaught)	ALL on maintenance treatment period achieving complete remission with ant-HBV negative (44)	NA	GenHevac B (Pasteur Connaught) (44)
Christopher C. Porter	2004	USA	Influenza H1N1	ALL on maintenance treatment who had completed their last delayed intensification at least 4 weeks (20)	Healthy siblings (14) and healthy individuals (35)	Influenza H1N1 (20)
			Influenza H3N2			Influenza H3N2 (20)
			Influenza B			Influenza B (20)
TORBEN EK	2006	Sweden	Tetanus	ALL in complete remission and at 1 month or 6 months after cessation of treatment (31 SR = 6 IR = 16 HR = 9)	Healthy individuals (18)	Tetanus (31)
			Hib			Hib (31)
G. Calaminus	2007	German	Diphtheria	ALL within 3 to 12 months after the end of maintenance therapy (HR = 28, LR = 31)	NA	Diphtheria (59)
			Tetanus			Tetanus (59)
BIROL BAYTAN	2008	Turkey	HBV	ALL who received primary HBV vaccination before diagnosis were HBsAg negative and anti-HBs positive (34)	NA	NA
Anne Reilly	2010	USA	H1N1	ALL on induction (8), postinduction (9), and maintenance phase (51)	NA	Influenza H1N1 (68)
Elham Shahgholi	2010	Iran	Influenza H1N1	ALL in first remission and receiving maintenance therapy (32)	Healthy siblings (30)	Influenza H1N1 (32)
			Influenza H3N2			Influenza H3N2 (32)
			Influenza B			Influenza B (32)
Selin Aytac	2010	Turkey	Measles	ALL on cessation of treatment (0–6, 6–12, 12–24, and more than 24 months after cessation) (77)	NA	Measles (45)
			Mumps	ALL on cessation of treatment (0–6, 6–12, 12–24, and more than 24 months after cessation) (76)	NA	Mumps (45)
			Rubella	ALL on cessation of treatment (0–6, 6–12, 12–24, and more than 24 months after cessation) (76)	NA	Rubella (45)
Thomas Lehrnbecher	2011	German	Diphtheria	Non-high-risk ALL on cessation of treatment (3, 6, and 9 months after cessation) (24)	NA	Diphtheria (24)
			Tetanus			Tetanus (24)
			Hib			Hib (24)
			Poliovirus			Poliovirus (24)
T. Ronan Leahy	2013	Ireland	Influenza H1N1	Children above the age of 6 months diagnosed and on treatment for ALL (45)	NA	Influenza H1N1 (45)
Leili Koochakzadeh	2014	USA	Diphtheria	ALL were newly diagnosed (*n* = 15), on maintenance therapy (*n* = 15) and 1 (*n* = 15), 3 (*n* = 15), 6 (*n* = 15), 12 months (*n* = 15) after ending of therapy	Healthy siblings (90)	Diphtheria (85)
			Tetanus			Tetanus (85)
			Pertussis			Pertussis (85)
			Measles			Measles (85)
			Mumps			Mumps (85)
			Rubella			Rubella (85)
Myriam Onoratelli	2016	Argentina	Measles	ALL on cessation of treatment for 6 to 18 months (61)	NA	NA
			Tetanus			
			Rubella			
Ashraf E. Fouda	2018	Egypt	Measles	ALL on cessation of treatment for more than 12 months (57)	NA	Measles (22)
			Mumps			Mumps (22)
			Rubella			Rubella (22)
Jessica. Bate	2019	United Kingdom	PCV13	a: ALL during the third cycle of maintenance chemotherapy (*n* = 39), ALL on cessation of treatment for 4 weeks (*n* = 40), ALL on cessation of treatment for 6 months (*n* = 39)	NA	PCV13 (117)
Young Bae Choi	2019	South Korea	HBV	Newly diagnosed ALL (88)	NA	HBV (72)
Karina A	2020	Canada	PCV	ALL on cessation of treatment for 4–12 months (74)	Healthy siblings (78)	PCV (73)
			Pertussis			Pertussis (73)
			Tetanus			Tetanus (73)
			Varicella			Varicella (73)
Ersin Toret	2020	Turkey	Measles	Newly diagnosed ALL (46)	NA	NA
			Mumps			
			Rubella			
			Varicella			
			HAV			
			HBV			
Sarah Dorval	2021	Canada	PCV	ALL on maintenance therapy and cessation of treatment (Group 1 *n* = 32: boosters during maintenance and post chemotherapy. Group 2 *n* = 39: booster post chemotherapy only)	NA	PCV (71)
L. Wang	2021	China	HBV	Newly diagnosed ALL (547)	NA	NA
Prasanth Varikkattu Rajendran	2022	India	HBV	Newly diagnosed ALL who were HBsAg negative (125). Group I: Anti-HBs titers > 10 mIU/L). Group II: Anti-HBs titers < 10 mIU/L.	NA	Exclude: Hepatitis B immunoglobulin was given
Tolga İnce	2024	Türkiye	HAV	Newly diagnosed ALL (78)	NA	NA
			HBV			
			Rubella			

## Supplementary Materials

The following supporting information can be downloaded at: https://www.mdpi.com/article/10.3390/vaccines13060605/s1, Table S1: The results of the risk of bias assessment for each included article.

## References

[1] Hunger S.P., Mullighan C.G. (2015). Acute Lymphoblastic Leukemia in Children. N. Engl. J. Med..

[2] Smith M.A., Seibel N.L., Altekruse S.F., Ries L.A., Melbert D.L., O’Leary M., Smith F.O., Reaman G.H. (2010). Outcomes for children and adolescents with cancer: Challenges for the twenty-first century. J. Clin. Oncol..

[3] Malard F., Mohty M. (2020). Acute lymphoblastic leukaemia. Lancet.

[4] Arber D.A., Orazi A., Hasserjian R., Thiele J., Borowitz M.J., Le Beau M.M., Bloomfield C.D., Cazzola M., Vardiman J.W. (2016). The 2016 revision to the World Health Organization classification of myeloid neoplasms and acute leukemia. Blood.

[5] Escherich G., Horstmann M.A., Zimmermann M., Janka-Schaub G.E. (2010). Cooperative study group for childhood acute lymphoblastic leukaemia (COALL): Long-term results of trials 82, 85, 89, 92 and 97. Leukemia.

[6] Hunger S.P., Lu X., Devidas M., Camitta B.M., Gaynon P.S., Winick N.J., Reaman G.H., Carroll W.L. (2012). Improved survival for children and adolescents with acute lymphoblastic leukemia between 1990 and 2005: A report from the children’s oncology group. J. Clin. Oncol..

[7] Lama-Sherpa T.D., Shevde L.A. (2020). An Emerging Regulatory Role for the Tumor Microenvironment in the DNA Damage Response to Double-Strand Breaks. Mol. Cancer Res..

[8] Page M.J., McKenzie J.E., Bossuyt P.M., Boutron I., Hoffmann T.C., Mulrow C.D., Shamseer L., Tetzlaff J.M., Akl E.A., Brennan S.E. (2021). The PRISMA 2020 statement: An updated guideline for reporting systematic reviews. BMJ.

[9] Top K.A., Vaudry W., Morris S.K., Pham-Huy A., Pernica J.M., Tapiéro B., Gantt S., Price V.E., Rassekh S.R., Sung L. (2020). Waning Vaccine Immunity and Vaccination Responses in Children Treated for Acute Lymphoblastic Leukemia: A Canadian Immunization Research Network Study. Clin. Infect. Dis..

[10] Lehrnbecher T., Schubert R., Allwinn R., Dogan K., Koehl U., Grüttner H.P. (2011). Revaccination of children after completion of standard chemotherapy for acute lymphoblastic leukaemia: A pilot study comparing different schedules. Br. J. Haematol..

[11] Baytan B., Gunes A.M., Gunay U. (2008). Efficacy of primary hepatitis B immunization in children with acute lymphoblastic leukemia. Indian. Pediatr..

[12] Koochakzadeh L., Khosravi M.H., Pourakbari B., Hosseinverdi S., Aghamohammadi A., Rezaei N. (2014). Assessment of immune response following immunization with DTP/Td and MMR vaccines in children treated for acute lymphoblastic leukemia. Pediatr. Hematol. Oncol..

[13] Toret E., Yel S.E., Suman M., Duzenli Kar Y., Ozdemir Z.C., Dinleyici M., Bor O. (2021). Immunization status and re-immunization of childhood acute lymphoblastic leukemia survivors. Hum. Vaccines Immunother..

[14] Wang L., Hu H., Zhang R., Zheng X., Li J., Lu J., Zhang Y., Qi P., Lin W., Wu Y. (2021). Changes in the hepatitis B surface antibody in childhood acute lymphocytic leukaemia survivors after treatment with the CCLG-ALL 2008 protocol. Clin. Exp. Immunol..

[15] İnce T., Tüfekçi Gürocak Ö., Totur G., Yılmaz Ş., Ören H., Aydın A. (2024). Waning of Humoral Immunity to Vaccine-Preventable Diseases in Children Treated for Acute Lymphoblastic Leukemia: A Single-Center Retrospective Cross-Sectional Analysis. Turk. J. Haematol..

[16] Choi Y.B., Lee N.H., Yi E.S., Kim Y.J., Koo H.H. (2019). Changes in hepatitis B antibody status after chemotherapy in children with acute lymphoblastic leukemia. Pediatr. Blood Cancer.

[17] Rajendran P.V., Thankamony P., Rajeswari B., Sojamani G.C., Nair M., Parukuttyamma K., Krishna Km J. (2023). Loss of protective anti-HBs titers and seroconversion to hepatitis B vaccination in children during chemotherapy for acute lymphoblastic leukemia. Pediatr. Blood Cancer.

[18] Ogra P.L., Sinks L.F., Karzon D.T. (1971). Poliovirus antibody response in patients with acute leukemia. J. Pediatr..

[19] Lange B., Shapiro S.A., Waldman M.T., Proctor E., Arbeter A. (1979). Antibody responses to influenza immunization of children with acute lymphoblastic leukemia. J. Infect. Dis..

[20] Porter C.C., Edwards K.M., Zhu Y., Frangoul H. (2004). Immune responses to influenza immunization in children receiving maintenance chemotherapy for acute lymphoblastic leukemia. Pediatr. Blood Cancer.

[21] Shahgholi E., Ehsani M.A., Salamati P., Maysamie A., Sotoudeh K., Mokhtariazad T. (2010). Immunogenicity of trivalent influenza vaccine in children with acute lymphoblastic leukemia during maintenance therapy. Pediatr. Blood Cancer.

[22] Lange B., Jakacki R., Nasab A.H., Luery N., McVerry P.H. (1989). Immunization of leukemic children with Haemophilus conjugate vaccine. Pediatr. Infect. Dis. J..

[23] Yetgin S., Tunc B., Koc A., Toksoy H.B., Ceyhan M., Kanra G. (2001). Two booster dose hepatitis B virus vaccination in patients with leukemia. Leuk. Res..

[24] Calaminus G., Hense B., Laws H.J., Groeger M., MacKenzie C.R., Gobel U. (2007). Diphtheria (D) and tetanus (T) antibody values in children with acute lymphoblastic leukaemia (ALL) after treatment according to Co-ALL 05/92. Klinische Padiatrie.

[25] Aytac S., Yalcin S.S., Cetin M., Yetgin S., Gumruk F., Tuncer M., Yurdakok K., Gurgey A. (2010). Measles, mumps, and rubella antibody status and response to immunization in children after therapy for acute lymphoblastic leukemia. Pediatr. Hematol. Oncol..

[26] Leahy T.R., Smith O.P., Bacon C.L., Storey L., Lynam P., Gavin P.J., Butler K.M., O’Marcaigh A.S. (2013). Does vaccine dose predict response to the monovalent pandemic H1N1 influenza a vaccine in children with acute lymphoblastic leukemia? A single-centre study. Pediatr. Blood Cancer.

[27] Fouda A.E., Kandil S.M., Boujettif F., Salama Y.S., Fayea N.Y. (2018). Humoral immune response of childhood acute lymphoblastic leukemia survivors against the measles, mumps, and rubella vaccination. Hematology.

[28] Bate J., Borrow R., Chisholm J., Clarke S.C., Dixon E., Faust S.N., Galanopoulou A., Goldblatt D., Heath P.T., Maishman T. (2020). Thirteen-Valent Pneumococcal Conjugate Vaccine in Children with Acute Lymphoblastic Leukemia: Protective Immunity Can Be Achieved on Completion of Treatment. Clin. Infect. Dis..

[29] Dorval S., Gantt S., Leclerc J.M., Laverdière C., Ovetchkine P., Tapiéro B. (2021). Pneumococcal vaccination during chemotherapy in children treated for acute lymphoblastic leukemia. Pediatr. Blood Cancer.

[30] Tsang V. (2012). Vaccination recommendations for the hematology and oncology and post-stem cell transplant populations. J. Adv. Pract. Oncol..

[31] de de la Fuente Garcia I., Coïc L., Leclerc J.M., Laverdière C., Rousseau C., Ovetchkine P., Tapiéro B. (2017). Protection against vaccine preventable diseases in children treated for acute lymphoblastic leukemia. Pediatr. Blood Cancer.

[32] Anafy A., Gilad G., Michaan N., Elhasid R., Rosenfeld-Kaidar H., Arad-Cohen N., Cohen M.S., Shachor-Meyouhas Y., Grisaru-Soen G. (2023). Revaccination of children with acute lymphoblastic leukemia following completion of chemotherapy. Pediatr. Blood Cancer.

[33] Close E., McConnell G., Cross S., Bradford J.L. (2020). Immunogenicity of Childhood Vaccines after Pediatric Cancer. Am. Fam. Physician.

[34] van Tilburg C.M., Sanders E.A., Rovers M.M., Wolfs T.F., Bierings M.B. (2006). Loss of antibodies and response to (re-)vaccination in children after treatment for acute lymphocytic leukemia: A systematic review. Leukemia.

